# Simultaneous measurement of electroencephalography and near-infrared spectroscopy during voluntary motor preparation

**DOI:** 10.1038/srep16438

**Published:** 2015-11-17

**Authors:** Takuro Zama, Sotaro Shimada

**Affiliations:** 1 Electrical Engineering Program, Graduate School of Science and Technology, Meiji University, 1-1-1 Higashi-Mita, Tama-ku, Kawasaki-shi, Kanagawa 214-8571, Japan; 2 Department of Electronics and Bioinformatics, School of Science and Technology, Meiji University, 1-1-1 Higashi-Mita, Tama-ku, Kawasaki-shi, Kanagawa 214-8571, Japan

## Abstract

We investigated the relationship between electrophysiological activity and haemodynamic response during motor preparation by simultaneous recording of electroencephalography (EEG) and near-infrared spectroscopy (NIRS). It is still unknown how exactly EEG signals correlate with the haemodynamic response, although the activation in the premotor area during motor preparation has been captured by EEG and haemodynamic approaches separately. We conducted EEG-NIRS simultaneous recordings over the sensorimotor area with a self-paced button press task. Participants were instructed to press a button at their own pace after a cue was shown. The result showed that the readiness potential (RP), a negative slow potential shift occurring during motor preparation, on C3 in the extended 10–20 system occurred about 1000 ms before the movement onset. An increase in concentration of oxyhaemoglobin (oxyHb) in the premotor cortex during motor preparation was also confirmed by NIRS, which resulted in a significant correlation between the amplitude of the RP and the change in oxyHb concentration (Pearson’s correlation *r*^2^ = 0.235, *p* = 0.03). We show that EEG-NIRS simultaneous recording can demonstrate the correlation between the RP and haemodynamic response in the premotor cortex contralateral to the performing hand.

Recent advanced functional neuroimaging studies have used simultaneous recording with multiple modalities^1^,^2^,^3^,^4^,^5^,^6^,^7^,^8^,^9^,^10^,^11^. Noninvasive neuroimaging methods can be divided into two groups based on their measurement principles: electrophysiological measurement, such as electroencephalography (EEG) and magnetoencephalography (MEG), and haemodynamic measurement, such as functional magnetic resonance imaging (fMRI) and near-infrared spectroscopy (NIRS). These modalities suffer from particular resolution limits: electrophysiological methods suffer in terms of spatial resolution, while haemodynamic methods suffer from the slow vascular response limiting their temporal resolution^2^. The multi-modal approach can be productive in conquering these limits by complementing each signal^12^,^13^,^14^. Sato *et al.*^8^ proposed variational Bayesian multimodal encephalography (VBMEG) as a hierarchical Bayesian estimation method, and combination of MEG and fMRI signals confirmed that VBMEG could be used to estimate brain activity with a high spatiotemporal resolution. Another study has shown the improvement of current source estimation of EEG by incorporating prior fMRI information^1^; they coupled the EEG signal with the BOLD signal recorded in advance with the same experimental paradigm. Although these studies use signals acquired in separate (fMRI and EEG/MEG) experiments, it is obviously better to couple signals acquired from the simultaneous measurement of two modalities.

While fMRI is difficult to apply concurrently with EEG/MEG, NIRS is suited to simultaneous recording with electrophysiological measurement because it uses near-infrared light, and electrophysiological measurements are not contaminated by light. Furthermore, owing to its lower cost, tolerance to participants’ motion artifacts and higher portability, EEG-NIRS simultaneous recording has a high compatibility with long-term recording, infant and patient studies, and naturalistic human motor control studies. EEG-NIRS simultaneous long-term recording can further investigate interictal epileptic discharges (IEDs) or seizures^5^,^7^,^9^,^11^, using haemodynamic changes time-locked to IEDs. The coupling of EEG and NIRS reveals the dynamic focal and remote haemodynamic changes of seizures^5^,^7^ and can detect a preictal state existing about 5 min prior to the seizure^9^. Co-registration of EEG and NIRS is also suitable to investigate the linguistic functions of newborns and infants^6^,^10^,^15^, because this technique is completely silent and is tolerant to participants’ motion. The coupling of event related potentials (ERPs) and haemodynamic responses by NIRS has also been applied to studies of the visual cortex^3^,^4^. The tolerance to participants’ motion artifacts is also advantageous to studies of brain–machine (–computer) interfaces (BMI/BCI). BMI systems often use motor-related brain activity as a trigger to control the machine. Fazli *et al.*^2^ found that haemodynamic responses are useful in the enhancement of EEG-based BMI performance. Their “hybrid BCI” system^16^ consisted of an AND gate connecting the EEG frequency change detector and the NIRS haemodynamic response detector. The coupling of the electrophysiological signal and haemodynamic response has the potential to make the most of both the spatial and temporal resolutions. From the above, the major studies on EEG-NIRS simultaneous recording can be divided into three types depending on their purpose: i) the improvement of current source estimation with the haemodynamic response; ii) the monitoring of the haemodynamic response time-locked to the EEG signal; and iii) the investigation of the spatial/amplitude correlation between the EEG signal and the NIRS signal.

In EEG-NIRS simultaneous recording study of type (iii), the discovered signal correlation becomes a valid clue to robustly detect a certain neural process, such as action execution. However, few studies have investigated EEG-NIRS signals correlation during motor preparation. Previous studies^17^,^18^,^19^,^20^,^21^ have reported a feature in EEG reflecting motor preparation called Bereitschaftspotential or readiness potential (RP), which is a negative slow potential shift starting about 1–2 s before the voluntary motor movement. In particular, the RP on the contralateral motor area is superior in the amplitude compared to the ipsilateral motor area^17^. Other studies have shown that the contralateral premotor cortex is activated during motor preparation, which has been measured by fMRI/NIRS^22^,^23^,^24^,^25^,^26^,^27^. Thus, we hypothesized that RP and the haemodynamic response are negatively (note that RP has negative value) correlated in the premotor cortex contralateral to the performing hand during motor preparation. However, the relationship between the RP and the haemodynamic response has not been fully investigated^28^. The NIRS signal, which has a higher S/N ratio than the EEG and a higher temporal resolution than the fMRI, may have potential to complement EEG signal (RP) for further investigation of the neural processes of motor preparation. In this study, we performed EEG-NIRS simultaneous recording during motor preparation to see whether RP and the haemodynamic response are negatively correlated in the contralateral premotor area.

## Results

**Figure 1:**
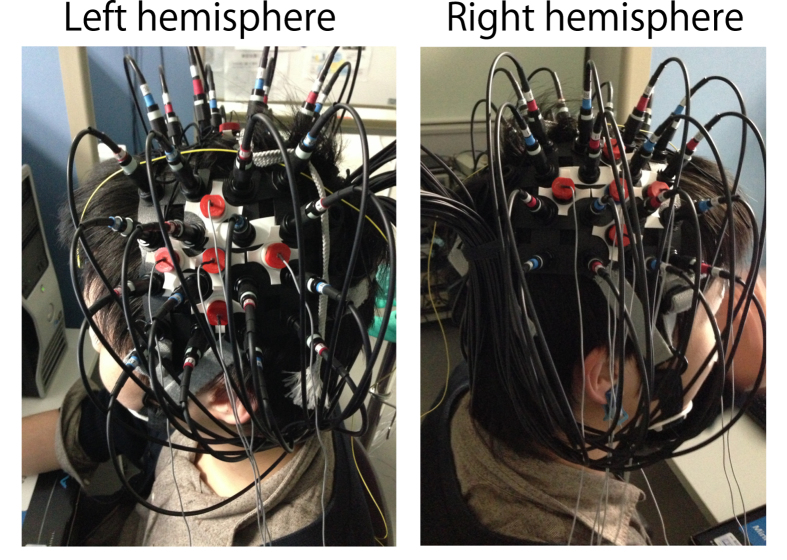
Customised EEG-NIRS cap. The cap held 16 NIRS optodes for each hemisphere and EEG electrodes were placed crosswise around C3/C4.

**Figure 2:**
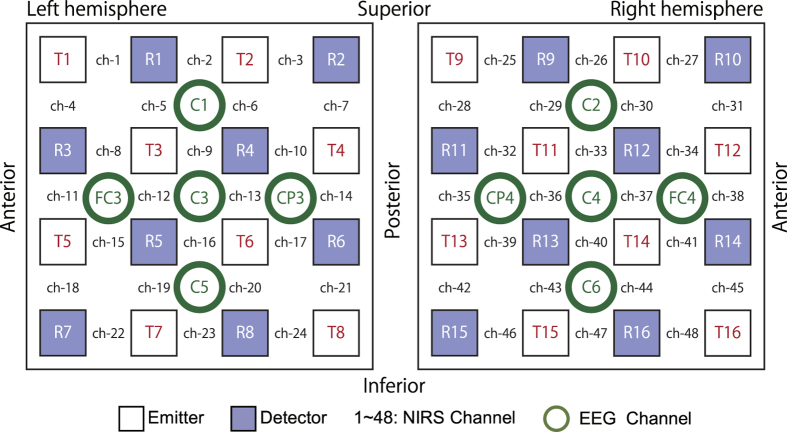
The location of the near-infrared spectroscopy (NIRS) optodes and electroencephalography electrodes placed on the motor area in both hemisphere. The distances between each NIRS emitter and the corresponding detector and between each EEG electrode were set at 3 cm. C3/C4 were located at the centre of each lattice.

Sixteen participants performed a self-paced button press task with inter-trial intervals of 17 s. They completed 35 sessions of the voluntary button press task with their right index finger. We simultaneously recorded the EEG and the NIRS using a customised EEG-NIRS cap (Fig. 1). We defined the C3 electrodes (centre of motor area; Fig. 2) as the ROI and detected the onset of the RP with the same method as the previous study^20^. We also examined C4 electrode as the control site.

**Figure 3:**
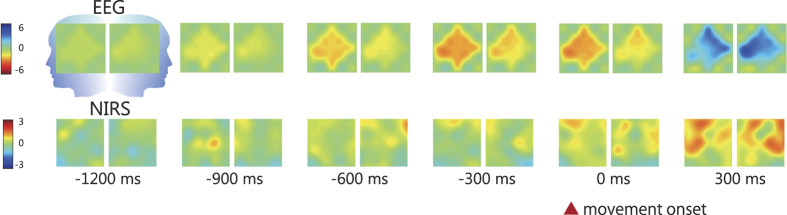
The sequential topological activation maps of changes in EEG (μV) and oxyHb (z-value) during the preparation of self-paced movement. EEG and NIRS signals were averaged across 15 participants. The location of optodes and electrodes is depicted in Fig. 2. This figure was illustrated by T. Z.

**Figure 4:**
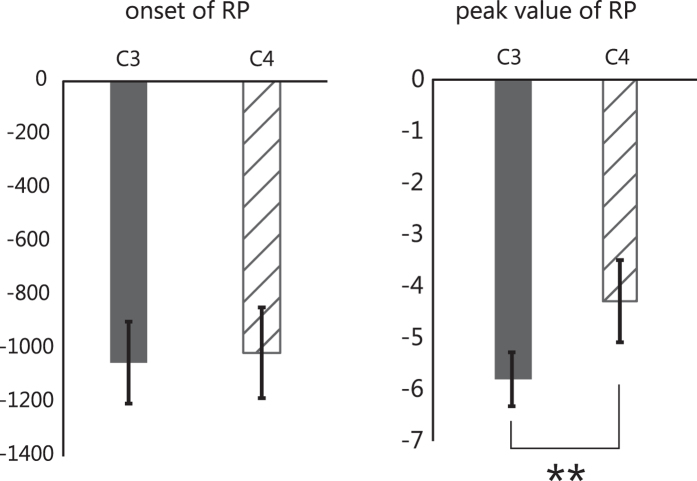
Results of the individual RPs on C3 and C4. Error bars indicate standard error. (**a**) The average onset of RP for each hemisphere. No significant difference in the onset of RP was found (*t*(14) = −0.63, *p* = 0.54). (**b**) The average peak-value of RP on each hemisphere. RPs in the contralateral cortex were significantly larger than those in the ipsilateral cortex (*t*(14) = −3.46, *p* = 0.004).

Figure 3 shows sequential topological activation maps of changes in EEG and oxyhaemoglobin (oxyHb) during motor preparation. The mean onset of the RP on C3 was −1050 ± 153 (mean ± s.e.m.) ms and that on C4 was −1013 ± 169 ms. The onset of the RP was not significantly different between C3 and C4 (*t*(14) = −0.63, *p* = 0.54, two-tailed one sample t-test; Fig. 4 left). The peak value of the RP on C3 was −5.80 ± 0.52 (mean ± s.e.m.) μV and that on C4 was −4.29 ± 0.80 μV. The peak value of the RP on C3 was significantly larger than that on C4 (*t*(14) = −3.46, *p* = 0.004, two-tailed one sample t-test; Fig. 4 right). Namely, the RPs occurred bilaterally at around −1000 ms and showed contralateral (left) cerebral dominance (C3 > C4).

Using sliding window analysis on the haemodynamic response (for details see the section **NIRS data analysis**), the oxyHb was found to be significantly started to increase in the contralateral premotor cortex (BA6, ch-8: [−41, 11, 60]) at about −900 ms (*t*(14) = 3.84, *p* = 0.0009). Although the right premotor area was also activated in ch-34 ([40, 10, 61], corresponded to the ch-8), the increase in oxyHb started just before the movement (at about −100 ms; *t*(14) = 3.79, *p* = 0.001). During the motor preparation, the contralateral premotor area was activated subsequently to the onset of the RP.

**Figure 5:**
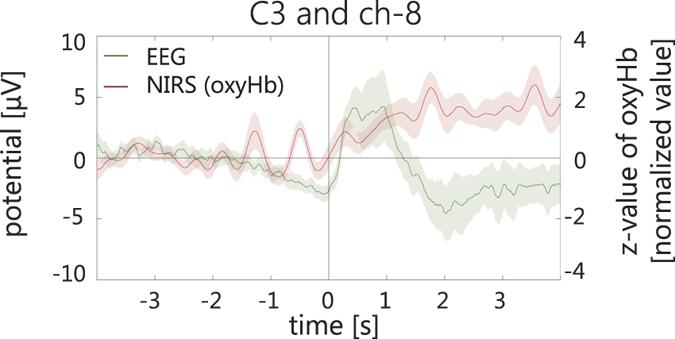
The grand averaged waveforms of the haemodynamic responses in the contralateral premotor area where the oxyHb (z-value) significantly increased during motor preparation were superimposed on the RP on C3. The zero second represents the movement onset. The pale colour areas show the standard error for each signal.

**Figure 6:**
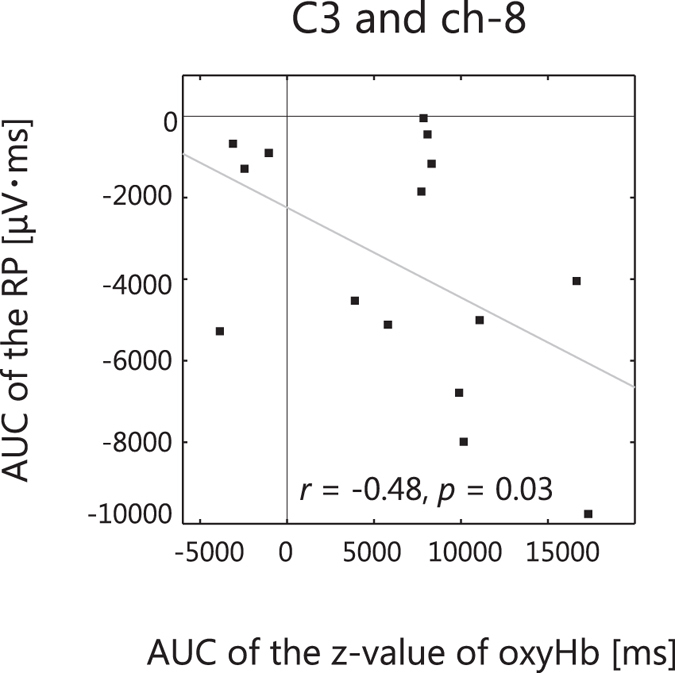
Scatter plot of areas under the curves (AUCs) of oxyHb and RP. There was a significant negative correlation between the change in RP on C3 and the change in oxyHb on ch-8 (Pearson’s correlation coefficient: *r*^2^ = 0.235, *p* = 0.03).

Figure 5 shows the grand averaged waveforms of the haemodynamic responses superimposed on the RP on C3 during motor preparation. With our strong assumption that the change in EEG signal correlated with the change in haemodynamic response in the left premotor cortex during motor preparation, we further conducted the correlation analysis between EEG and NIRS signals in the left premotor cortex (ch-8). The result showed that the increase in oxyHb in ch-8, in which the signal change was most prominent in the premotor area, was significantly correlated with the change in the RP on C3 (one-tailed Pearson’s rank correlation *r*^2^ = 0.235, *p* = 0.03; Fig. 6). Separately from the ROI analysis based on our hypothesis, we also tested the correlation between the EEG and NIRS in the right premotor area within the same time bin as the left premotor area. No such correlation was found in the right hemisphere (*r*^2^ = 0.0004, *p* = 0.53; C4 and ch-34). We also tested the interhemispheric difference of correlation coefficient between C3 and C4, which resulted in a marginal difference (left > right, *z* = 1.35, *p* = 0.09, one-tailed z test).

## Discussion

The present study investigated whether the haemodynamic response correlates with electrophysiological signals during motor preparation. RPs occurred bilaterally and the amplitude became larger over the contralateral region as the time approximated to the onset of movement. The result showed that the RP on C3 occurred about 1000 ms before the movement onset. As for the haemodynamic response, an increase in oxyHb mainly occurred the contralateral premotor cortex during motor preparation. Furthermore, we found that the magnitude of the change in oxyHb in the premotor cortex contralateral to the performing hand was correlated with that of the RP.

Our results of RP and oxyHb changes in the left motor area are consistent with the activity pattern found in previous studies. It has been shown that the RP can be divided into the early phase (early RP) and late phase (late RP)^17^. The early RP occurs in the supplementary motor area and, shortly thereafter, the lateral premotor cortex bilaterally^29^. The late RP starts about 400 ms before the movement onset in the premotor cortex and the M1, mainly contralaterally^17^,^21^,^30^,^31^,^32^. Libet *et al.*^20^ reported that the early RP on C3 started about 1000 ms before the self-paced movement. They postulated that the late RP reflects the cerebral volitional process uniquely involved in initiating a voluntary, fully endogenous action and that the early RP reflects a more general preparation or intention. Our result supports Libet’s hypothesis because the (early) RP on C3 occurred about 1000 ms before the movement onset with the self-paced voluntary button press task. Several studies also reported that the RP onset was about 1000 ms prior to the self-paced movement onset^18^,^19^,^21^. The larger peak amplitude of contralateral RP than that of ipsilateral RP is consistent with previous studies that reported lateralised distribution with maximum amplitude over the contralateral motor area^17^,^33^,^34^. The late RP is extremely confined to the region corresponding to the motor part of the primary sensorimotor cortex and the premotor cortex^30^,^35^,^36^. Our RP results confirmed those of previous studies, which reported that RPs occurred bilaterally at around −1000 ms and showed contralateral (left) cerebral dominance (C3 > C4). With respect to the haemodynamic response, the concentration of oxyHb increased in the contralateral premotor area. This result conforms to the previous fMRI studies^22^,^23^,^24^,^25^,^37^. Simon *et al.*^37^ reported activation in the contralateral premotor cortex during motor preparation for a key press task with the right index and middle fingers.

The remarkable result is the strong correlation between the magnitudes of the change in oxyHb and the change in the RP in the contralateral premotor cortex during motor preparation. Mosso^38^ and Roy *et al.*^39^ reported that neural activity is accompanied by a haemodynamic response. This coupling between the neural activity and the haemodynamic response is called neurovascular coupling. The correlation between the electrophysiological signal and the haemodynamic response has been also reported by simultaneous recording with EEG and NIRS. Takeuchi *et al.*^40^ investigated the correlation between somatosensory evoked potential (SEP) and the change in concentration of oxyHb with stimulation of the right median nerve. The change in concentration of oxyHb and the SEP occurred in the parietal association area contralateral to the stimulus. Furthermore, the distribution of the changes in oxyHb was similar to that of the current source density (CSD) of P22, which is an ERP constituting of the SEP. They found a significant spatial correlation between the CSD map and NIRS distributions, suggesting that electrophysiological activity underlying P22 induced haemodynamic responses. Another previous study also showed that the amplitude of the NIRS signal in Wernicke’s and Broca’s areas correlated with the N400 component of the EEG signal recorded during a semantic processing task^41^. Näsi *et al.*^4^ showed that the area under the curve (AUC) of the haemodynamic response correlated with the AUC of the visual evoked potential, which occurred using a pattern-reversing checkerboard stimulus. These previous studies reported a correlation between the EEG and NIRS signals as a response to external stimuli. Regarding spontaneous brain activity, the signal correlation has not been investigated during motor preparation, although EEG-NIRS simultaneous measurements have been reported with the RP paradigm^28^. In the present study, we showed a correlation between the EEG and NIRS signals during motor preparation over the contralateral motor areas.

Few studies have investigated the relationship between the electrophysiological signal and the haemodynamic response during motor preparation, although the contralateral premotor cortex has been suggested as the source of the RP^21^. The present study found a correlation between the EEG signal and the NIRS signal in the contralateral (left) premotor cortex during motor preparation with consideration of the delay of haemodynamic response. Our result of the specific correlation of RP with the haemodynamic response in the premotor area strongly suggests that the NIRS signal reflects the process of electric excitation^4^,^21^,^40^,^41^. Although, this result should be taken cautiously as the correlation does not necessarily mean that EEG and NIRS recorded the same neural activity. We suggest that EEG-NIRS simultaneous recording can show the correlation between the EEG and NIRS signals during motor preparation, which can be informative for increasing the S/N ratio of feature-related motor preparation.

## Methods

### Participants

Sixteen healthy volunteers (1 female, mean age 21.6, s.d. ± 0.7, range 20–23) participated in the experiment. One participant was removed from the analysis because EEG data of the participant showed no RP (for details see section **EEG data analysis**). Written informed consent was obtained from all participants. The experiments were approved by the ethics committee of the School of Science and Technology, Meiji University, and conducted according to the principles and guidelines of the Declaration of Helsinki.

### Procedure

Participants were seated on a comfortable chair in front of a 23-inch liquid-crystal colour monitor. The viewing distance was approximately 85 cm. The participant put their right arm on an armrest cushion set on the desk and put their index finger on the button (Serial Response Box, 200a, Psychology Software Tools, Inc., USA). The participant was instructed to watch the monitor throughout the experiment.

The experimental design consisted of 35 trials of a simple voluntary button press with inter-trial intervals of 17 s. The trial began with a white fixation cross that appeared at the centre of the black screen. The participants were then allowed to tap the button with their index finger in their own time. After the button was pushed, the fixation cross disappeared and the screen turned black (rest period). The participants were instructed to press the button forcefully and not to blink immediately before and after the button press.

### Simultaneous recording

Simultaneous measurements of EEG and NIRS were performed throughout the experiment. EEG electrodes and NIRS optodes were arranged into a customised EEG-NIRS cap (Fig. 1) with an inter-optode distance of 3 cm. The cap was customised based on a commercially available NIRS cap (Shimadzu, Japan) that had a 4 × 4 node grid for each hemisphere. EEG electrodes were installed in the gaps in the vertical and horizontal lattice containing the NIRS optodes. With this arrangement, 16 optodes and five electrodes constituted 24 NIRS and 5 EEG channels, respectively, for each hemisphere over the motor area (Fig. 2). The central EEG electrode was placed on C3 and C4 of the extended 10–20 system: namely, EEG signals were recorded at 10 channels (C1, FC3, C3, CP3, C5, C2, FC4, C4, CP4, and C6).

### EEG data acquisition

The EEG data were recorded using a 24-bit biosignal amplification unit (g.USBamp, g.tec Medical Engineering GmbH, Austria) at a sampling frequency of 256 Hz. The signals were recorded with active Ag/AgCl electrodes; an amplifier was encased within each electrode. The ground electrode was located on the forehead; the reference was mounted on the right earlobe. With the same amplification unit as the EEG, vertical electrooculography (EOG) was recorded from above and below the right eye and electromyography (EMG) was recorded in the right flexor digitorum superficialis muscle. The EEG and EOG were processed with a band-pass filter of 0.1 Hz to 30 Hz. The EMG was processed with a band-pass filter of 2 Hz to 100 Hz. A notch filter of 50 Hz was applied in all signals.

### NIRS data acquisition

Haemodynamic responses, which are temporal changes in concentrations of oxyhaemoglobin (oxyHb), deoxyhaemoglobin (deoxyHb) and total-haemoglobin (totalHb) in bilateral motor cortices, were assessed using a multichannel NIRS unit operating at 780, 805 and 830 nm wavelengths (OMM-3000, Shimadzu, Japan). The sampling frequency was 10 Hz.

The locations of optodes were measured using a 3D magnetic space digitiser (Fastrak, Polhemus, USA) to estimate the anatomical brain region beneath the NIRS channels. The measured position data were processed using a probabilistic spatial registration method^42^, which is available online (http://www.jichi.ac.jp/brainlab/tools.html). This method generates a probabilistic mapping between a NIRS channel and its corresponding anatomical brain region, which can be used for the interpretation of NIRS activation data.

### EEG data analysis

The Jade independent components analysis (ICA) was conducted on the EEG data to eliminate ocular artifacts. ICA components with the most significant correlations with the vertical EOG (*r*^2^ > 0.16) were rejected. The remaining data were back-projected to create EEG signals free from ocular artifacts. The EEG data were segmented into 17 s epochs: 5 s preceding and 12 s following the movement onset detected by EMG onset. The onset of the movement was decided based on the EMG data. The differential waveform of EMG was calculated. The onset of the movement was defined as the time when the differential waveform of EMG was over the three-sigma limits. Outlier trials were excluded from analysis according to the following steps: (1) we determined a threshold for the artifact as ± 60 μV; (2) the epochs where EEG signals of more than 3 electrodes exceeded the threshold before movement onset were selected; (3) the selected trial that was also visually recognised as an outlier by the experimenter was excluded from the further analysis^43^,^44^. Consequently, the number of trials submitted to the analyses per participant was 30.6 ± 3.9 (mean ± s.d.). The noise-free epochs were averaged and baseline-corrected using the time bin from −2200 ms to −2000 ms as a baseline.

The RP has been reported to be maximal over the contralateral central area^17^. In the central area, the C3/C4 is considered to be located on the functional region of hand representation in the primary motor area (M1)^45^. We defined C3 as the ROI to evaluate the same electrode as the previous study, using the same method to determine RP onset. We also analysed the RP on C4 as the control site to simply verify the laterality of the RP that have been shown by previous studies^17^. The onset of the RP was determined from the EEG waveform by the same method as that used in the previous study^20^. This method was based on the AUC of the RP. Integrated values of the RP curve were computed within a 100 ms long sliding-window with a 50% overlap. The sliding-window analysis was applied from 2000 ms before the movement onset (equal to the end of the baseline) to the onset of movement. The window raised a confirmation flag when an integrated value within the window exceeded −50 μV·ms in the negative direction; it should be noted that the integrated value takes a negative value if the RP occurs. If integrated values within the following sections continuously exceeded the threshold until the onset of movement, the onset of RP was decided as the time at which the flag was first raised. The onset of RP was examined with visual inspection by two researchers^30^,^31^. One participant was removed from the analysis because the EEG data of the participant showed no RP.

### NIRS data analysis

The NIRS signals were upsampled to 256 Hz with linear interpolation to have the same sampling frequency as the EEG so that the signals could be compared at the same time. A low-pass filter of 2 Hz and a high-pass filter of 0.02 Hz (zero phase each) were applied to the NIRS signals. The NIRS data were segmented into the same epoch of EEG signals. The epochs were baseline-corrected using the time bin from 4000 ms to 2000 ms before the onset of movement as a baseline. The averaged NIRS signals were normalised to z-values using s.d. during baseline to enable the comparison among participants and channels^46^.

We analysed the concentration change in oxyHb as the indicator of brain activity because the change in oxyHb is considered to be the most sensitive parameter of changes in regional cerebral blood flow, providing the strongest correlation with the BOLD signal among the three NIRS parameters^47^,^48^. We selected the channel in the premotor area that showed singnificant activation during motor preparation, survived after Bonferroni correction for multiple comparison, as the ROI. The effect size was calculated by averaging the z-value within a 4000 ms long sliding-window to compensate for the delayed rise time of the haemodynamic response^48^,^49^,^50^. The window was slid with a 100 ms step. The group analysis used a one-tailed one-sample t-test applied to the individual effect size data.

### Correlation analysis

Based on a strong *a priori* hypothesis that the amplitude of the RP and haemodynamic response in the left premotor area are negatively correlated, we carried out one-tailed Pearson’s correlation analysis between the amplitude of RP on C3 and that of the haemodynamic response in the most prominent channel (ch-8). To assess the oscillation of the RP, a zero-point correction was carried out for the RP by using the EEG potential at the time when the RP occurred. The integral of the area under the RP curve was calculated within the time bin from the RP onset (group-averaged) to the movement onset. The oxyHb also underwent a zero-point correction based on the increasing onset of each channel. Note that the AUC can be negative if EEG potential is negative. The integral of the area under the z-value curve was calculated within the time bin from the zero-point whose length was same as that of the sliding-window (4 s) in the “**NIRS data analysis**” section. The integrated values of the area under the EEG/NIRS curve were submitted to one-tailed Pearson’s correlation analysis.

## Additional Information

**How to cite this article**: Zama, T. and Shimada, S. Simultaneous measurement of electroencephalography and near-infrared spectroscopy during voluntary motor preparation. *Sci. Rep.* **5**, 16438; doi: 10.1038/srep16438 (2015).
